# Motor imagery EEG signal classification with a multivariate time series approach

**DOI:** 10.1186/s12938-023-01079-x

**Published:** 2023-03-23

**Authors:** I. Velasco, A. Sipols, C. Simon De Blas, L. Pastor, S. Bayona

**Affiliations:** 1 Department of Computer Science and Statistics, Rey Juan Carlos University, Madrid, Spain; 2 Department of Applied Mathematics, Science and Engineering of Materials and Electronic Technology, Rey Juan Carlos University, Madrid, Spain; 3 Center for Computational Simulation, Universidad Politecnica de Madrid, Madrid, Spain

**Keywords:** EEG, Classification, Multi-resolution, Multi-variate time series, Discrete Wavelet Transform (DWT)

## Abstract

**Background:**

Electroencephalogram (EEG) signals record electrical activity on the scalp. Measured signals, especially EEG motor imagery signals, are often inconsistent or distorted, which compromises their classification accuracy. Achieving a reliable classification of motor imagery EEG signals opens the door to possibilities such as the assessment of consciousness, brain computer interfaces or diagnostic tools. We seek a method that works with a reduced number of variables, in order to avoid overfitting and to improve interpretability. This work aims to enhance EEG signal classification accuracy by using methods based on time series analysis. Previous work on this line, usually took a univariate approach, thus losing the possibility to take advantage of the correlation information existing within the time series provided by the different electrodes. To overcome this problem, we propose a multivariate approach that can fully capture the relationships among the different time series included in the EEG data. To perform the multivariate time series analysis, we use a multi-resolution analysis approach based on the discrete wavelet transform, together with a stepwise discriminant that selects the most discriminant variables provided by the discrete wavelet transform analysis

**Results:**

Applying this methodology to EEG data to differentiate between the motor imagery tasks of moving either hands or feet has yielded very good classification results, achieving in some cases up to 100% of accuracy for this 2-class pre-processed dataset. Besides, the fact that these results were achieved using a reduced number of variables (55 out of 22,176) can shed light on the relevance and impact of those variables.

**Conclusions:**

This work has a potentially large impact, as it enables classification of EEG data based on multivariate time series analysis in an interpretable way with high accuracy. The method allows a model with a reduced number of features, facilitating its interpretability and improving overfitting. Future work will extend the application of this classification method to help in diagnosis procedures for detecting brain pathologies and for its use in brain computer interfaces. In addition, the results presented here suggest that this method could be applied to other fields for the successful analysis of multivariate temporal data.

**Supplementary Information:**

The online version contains supplementary material available at 10.1186/s12938-023-01079-x.

## Background

One of the big challenges in the XXI century, as an essential part of human brain analysis procedures, is the determination of mathematical models capable to explain and forecast the relationships between human activities and electroencephalography (EEG) signals. EEG signals produce data organized in temporal sequences with a structured behavior and have been used for different purposes, from seizure detection and epilepsy diagnosis [1–4], to automatic detection of abnormal EEG [5–8], and recognition of Alzheimer’s disease brain activity [9], the detection of awareness [10], or the use of brain–computer interfaces (BCI) [11, 12]. All these works require addressing the complexity of obtaining high-accuracy EEG classification, which is the goal of our paper.

In particular, we will study and apply time series analysis methods to achieve high accuracy in the classification of motor imagery EEG signals. However, we believe that there is still room for improvement in the classification and interpretability of the factors and variables that influence the model, as well as improvements in computational efficiency. The classification approach will be proved on consciousness-related data. When it comes to detecting consciousness in patients with disorders of consciousness (DOC), a large number of patients diagnosed as vegetative present a certain level of consciousness when judged by experienced professionals, demonstrating the difficulty of this task. One of the most commonly used techniques consists in visualizing brain activity through fMRI while the subject performs certain mental tasks. However, performing fMRI on this type of patients is very expensive, dangerous, and in many cases impractical due to metal implants.

The recording of brain activity using EEG signals and its subsequent characterisation, especially for the study of consciousness, has therefore become a trending topic, as this technology solves several of the problems associated with fMRI and has been shown to be able to produce reliable results [13]. These studies depend strongly on clinical trials, causing deficiencies in clinical robustness inference due to their limited sample representativeness. Recent advances in EEG signal analysis, based on brain images, can detect capacity reactions and activity in non-reactive patients with disorders of consciousness. Furthermore, some of these techniques have shown brain activity in clinical trials in comatose patients similar to that of healthy subjects which could be useful in the diagnosis and monitoring of the patient’s progress. In this line, Henriques et al. propose the design of an action protocol based on EEG trials regarding the consciousness level of a given patient to forecast the awareness level (based on registered EEG signals of patients imagining hand and feet movements) [10]. We will examine whether it is possible to improve the classification of the data in this study and to provide information about the most relevant features.

Another field where EEGs have great prominence is the field of BCI. The idea is to capture certain brain processes and send this information to a computer for interaction purposes, to control robotic devices, or for entertainment applications [14]. Currently, there are two main approaches to develop these BCI. One of them is based on evoked potentials where the patient responds to certain stimuli, and this response is captured and processed by the BCI triggering a certain action. Here, the patient cannot trigger actions unilaterally as it is necessary that the stimulus that triggers the response is first produced. In the other approach, the one that concerns us, the BCIs are based on spontaneous signals (normally based on motor tasks) produced by the person [15]. These tasks can be real or imaginary but there are many similarities between them [16]. A process of feature extraction is required for the BCI system to interpret and classify EEG data.

The time series analysis is one of the most successful techniques on account of the temporal structural nature of the data. For example, nonlinear time series analysis was proposed to provide new and Additional file about the epileptogenic process, improving presurgical evaluation [17]. In 2005, Kannathal et al. applied it to EEG signals [18]. Other relevant work along this line include the classification of EEG signals in binary groups by means of standard artificial neural networks to discriminate between normal or epileptic individuals [19], the use of a cross-correlation based feature extractor aided with a support vector machine classifier for emotional speech recognition [20], or the use of wavelets for a diagnostic tool for Alzheimer’s disease [21]. Some works developing general adaptive methods are based on weighted-distance nearest-neighbor classifiers [22], the Discrete Wavelet Transform (DWT) based feature extraction schemes [23], and multi-trial EEG clustering [24].

Specifically for EEG classification for BCI, some approaches are based on deep learning. For example, Gao et al. constructed a convolutional neural network with long short-term memory (CNN-LSTM) framework, which allows extracting the spectral, spatial, and temporal features of EEG signals, to achieve the high classification accuracies of Steady State Motion Visual Evoked Potential SSMVEP-based Brain Computer Interface (BCI) signals [25]. Anwar and Eldeib proposed a method that investigates a multi-class classification problem with AlexNet CNN and topographic images as features [26]. Sundaresan et al. investigated the feasibility of exploiting electroencephalography (EEG) signals for stress assessment by comparing several ML classifiers as support vector machine (SVM) and deep learning methods [27]. Also, Xie et al. developed classification methods incorporating transformer models when considering deep-network methods for EEG classification [28]. Regarding motor imagery BCIs, Tibrewal et al. studied the benefits of deep learning in improving the performance for different user groups [29].

Other BCI applications are spellers, such as the work in [30] for P300 speller detection, or the interesting review found in [12]. Classification of EEG signals by machine learning methods such as support vector machines can be found in [31]. Khare and Bajaj used wavelet decomposition in [32], while Narim combined wavelet decomposition and neuronal networks [33]. Other approaches for classification of EEG are random forest [34], or transfer discriminative dictionary learning with label consistency [35], among others.

Methods based on Time Series Analysis have proven to be effective when applied to EEG data, however, most proposals currently use a univariate approach [36, 37], where different characteristics (such as autocorrelation, peridograms, or wavelet characteristics) can be used in standard discriminant analysis. These approaches are useful to know the characteristics of a time series of trend, cycle, seasonality, or to make predictions, but they do not take into account the interrelation between different variables (the variable under study with other relevant variables), and therefore their usefulness may be limited.

A complete review of the state-of-the-art for mutivariate time series classification can be found in [38]. Some works use deep learning based algorithms for multivariate classification purposes. Karim et al. proposed transforming the existing univariate time series classification models, the Long Short Term Memory (LSTM) for Multivariate Time Series Classification [39]. Both the works of Ismail et al. and that of Ruiz et al. recently proposed bespoke Multivariate Time Series Classification (MTSC) algorithms based on deep learning [40, 41]. Some authors have even worked on improving the interpretability of deep learning models applied to multivariate time series classification [42].

Within the field of BCI, Morabito et al. presented applications of deep learning approaches in brain engineering and biomedical signal processing [43] and Chen et al. [44], proposed a time-frequency deep metric learning model for multivariate time series classification.

An advantage of using multivariate instead of univariate classification is that you can capture cross-dependencies that are not considered in univariate classification, and by measuring the dissimilarity between multivariate time series, both the cross-dependence and the serial dependence are continuously captured [41]. The cross-autocorrelation function measures not only the strength of the relationship, but also its direction. This multivariate approach has been used to detect anomalies in time series in different fields such as health care, finance and meteorological analysis [45]. Previous work in multivariate time series classification is discussed in [46], who proposed a fuzzy classification model for classifying patterns and clustered time series based on their wavelet variances at different scales. Also, univariate and multivariate features, i.e. variances and wavelet correlations, were combined to classify multivariate time series in [47], both considering hierarchical and non-hierarchical classification approaches. More recent techniques were proposed by Mandic et al. [48] for multivariate signals suggesting the Multivariate Empirical Mode Decomposition (MEMD), capable to deal with unbalanced multichannel data and nonuniform sampling. Last, discriminant and wavelet analyses were used for multivariate classification of electrocardiography (ECG) signals in [49]. It should be pointed out that the time series generated from EEG and ECG signals differ substantially, as ECG patterns include depolarization of the atria (P wave), depolarization of the ventricles; and repolarization of the ventricles (T wave) patterns repeated each heart beating, whereas EEG patterns occurs on event-related potentials.

In particular, regarding feature extraction methods for classifying EEG, different approaches are based on time, frequency or time-frequency domains. Regarding the alternatives based on the time domain, some works are based on an extension of the autoregressive models [50, 51] or Hermite decomposition [52]. As for frequency-domain analysis of the EEG signals, multiple works are based on fast Fourier transform [53, 54]; or on power spectral density [55, 56]. These previous approaches are sometimes ineffective due to the lack of spectral or temporal characteristics. To overcome these problems, hybrid methods, known as time-frequency domain methods, have been developed. The most generalized way to implement this approach is to use the short-time Fourier Transform [57–59]; the continuous wavelet transform [60]; the DWT [61, 62]; the wavelet packet decomposition (WPD) [63], or the Common Spatial Pattern feature extraction method [64, 65]. It is interesting to note that of all these options for feature extraction, the techniques based on decomposing the signal, like DWT and WPD, are very effective because the information of EEG data is carried in different bands and these approaches can decompose the waves in different resolutions and scales [66]. Moreover, these techniques are able to extract dynamic features [67].

The present work builds on the work of Cruse et al. [13] and Henriques et al. [10] and focuses on presenting an accurate approach to classify EEG signals, in this case, applying it to a dataset obtained in different mental tasks to assess the level of consciousness of DOC patients. The methodology we propose in this work is based on DWT feature extraction. However, we consider a multivariate environment that allows us to extract features from the relationships between different electrodes and to evaluate the impact of the inherent relationships between them, unlike traditional univariate methods, which ignore these relationships. The results obtained substantially improve the results shown in [13, 10] using the same motor imagery EEG task dataset. Our model seeks not only to generate an accurate classification, but also to be explainable. Importantly, our model provides information on which features are most important, which could have clinical relevance. Despite this, in order to provide more information, we will also compare our results with deep learning techniques, like the approach proposed by Karim et al. [39] and the more interpretable representation based on the work of Baldan and Benitez [42] Noteworthy, the dataset, despite having been acquired to analyse consciousness, could be perfectly usable to differentiate between two different motor tasks in a BCI environment. To our knowledge, this multivariate classification methodology, which does not use deep learning, had never been applied to the BCI field and, given that the results have been highly accurate, we believe that the BCI field could also benefit from this type of analysis; however, further research should confirm this.

## Results

Here we present the results obtained by applying the classification algorithm described in "Methods" section to EEG data consisting of different exercises in which subjects move their hands or feet. Note that the classifier has been trained with the data of all subjects at the same time, thus being the results of a multi-subject classifier. EEG data were registered using a cap with 64 electrodes, although the eye electrode was ignored for classification purposes (for more information see subsection "Experimental protocol").

It should be noted that the proposed method has a number of parameters that may affect performance. These parameters are, on the one hand, those related to DWT such as the specific wavelet (or filter) to be used, or the total number of variables selected to be taken into account, while other parameters are more related to the data, such as the type of features to be calculated (variance, correlation or both) or the classification method used (linear or quadratic). For more information, please see "Methods" section.

**Fig. 1:**
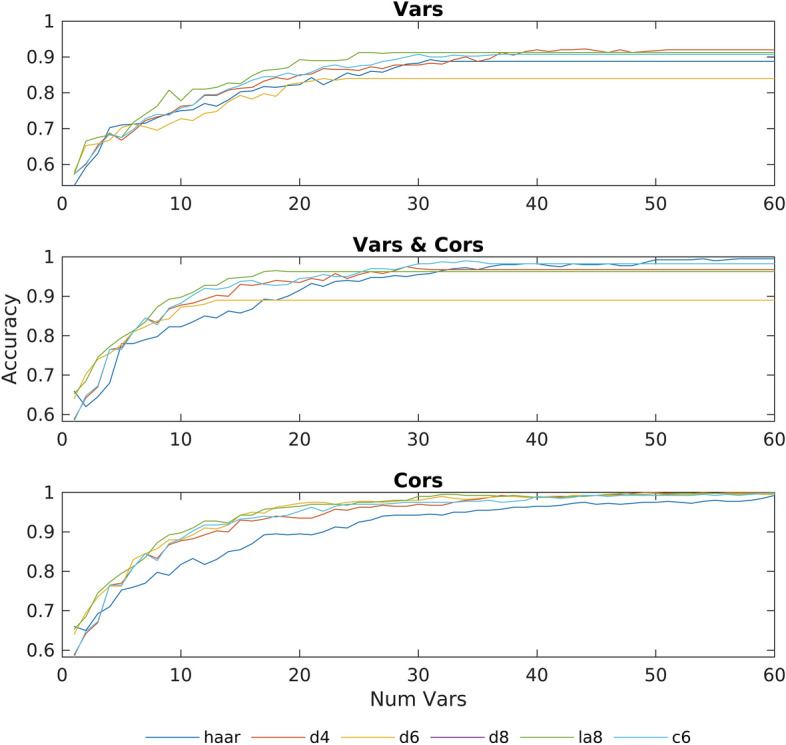
Accuracy obtained using a linear discriminant with subsets of most discriminant features containing between 1 and 60 variables

**Fig. 2:**
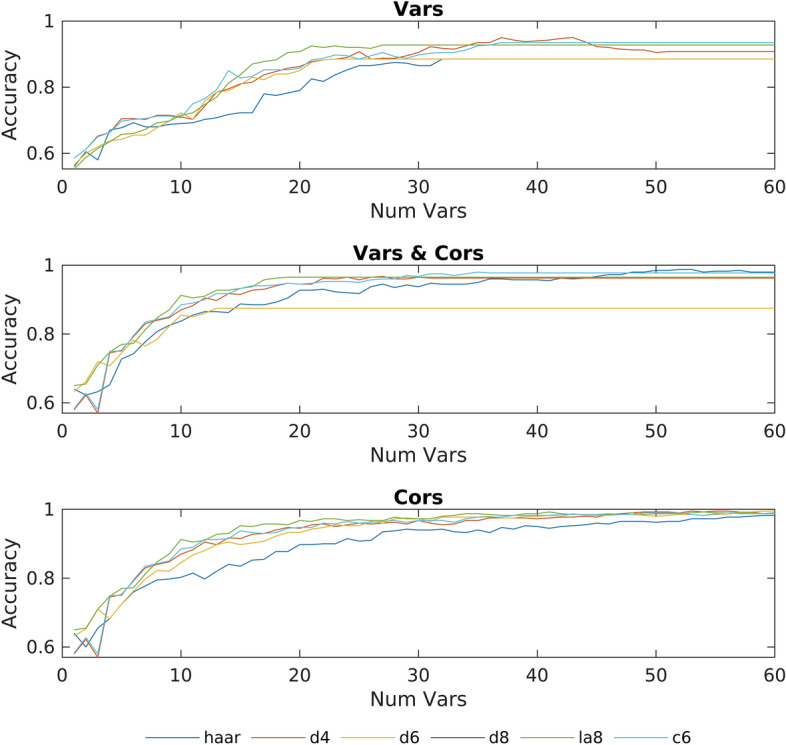
Accuracy obtained using a quadratic discriminant with subsets of most discriminant features containing between 1 and 60 variables

Different combinations of features and classification methods can affect the overall performance of the method, and also give us some clues about the behavior of the signals. Therefore, results have been generated for each possible combination of method parameters (filter, features, and number of variables) by running the complete method for each combination. This results can be seen in Figs. 1 and 2, where each figure shows the accuracy obtained by each of the 6 wavelets as a function of the variables taken into account. Each figure has three graphs, depending on whether the algorithm uses only the variances (Vars), only the correlations (Cors), or both variances and correlations (Vars & Cors). These graphs have been computed using either a linear or a quadratic discriminant.

As can be seen in Fig. 1, with a linear discriminant using 55 variables or more, a classification accuracy of 100% is obtained using the *d4* wavelet, although with 50 variables this same wavelet reaches results close to 100%, improving the results reported in [21]. Regarding the other wavelets examined, we can see how all of them, except for Haar, achieve an accuracy between 98% and 100% using 40 variables, while with 45 variables all of them exceed 99% of accuracy. On the other hand, if we compare the use of a linear discriminant versus the quadratic discriminant, we see that, for these data, the linear discriminant is more accurate. We can also observe that, in our experiment, the correlation between different electrodes is most important that the variances themselves, indicating that the activation relationships between the different electrodes are more important than the activation of the individual electrodes. In addition, we can also observe that the accuracy using both, correlations and variances, is lower than using only correlations. This effect may be due to a suboptimal selection of the set of features by the algorithm when selecting the most discriminating variables. As for the execution time, on a computer with a Ryzen 1600x and 16Gb of RAM, the process of checking the accuracy obtained for all combinations of possible parameters has taken 1 day. Moreover, it is important to emphasize that it is not necessary to use dedicated GPUs with specific memory requirements (in our algorithm RAM memory helps to increase execution speed, but you can sacrifice speed and reduce RAM usage if necessary). On the other hand, it must be noted that the execution time of 1 day is to obtain the optimal parameters for a particular problem. Once these parameters are selected, the algorithm only takes a few minutes to obtain the results. Another advantage is that the algorithm scales linearly with the number of variables considered, therefore, increasing the number of variables will not cause a drastic increase in the execution time. In addition, it is important to keep in mind that the classification method (linear or quadratic) does not significantly affect the execution time, because the execution time of this step is small when compared with the total execution time.

**Fig. 3:**
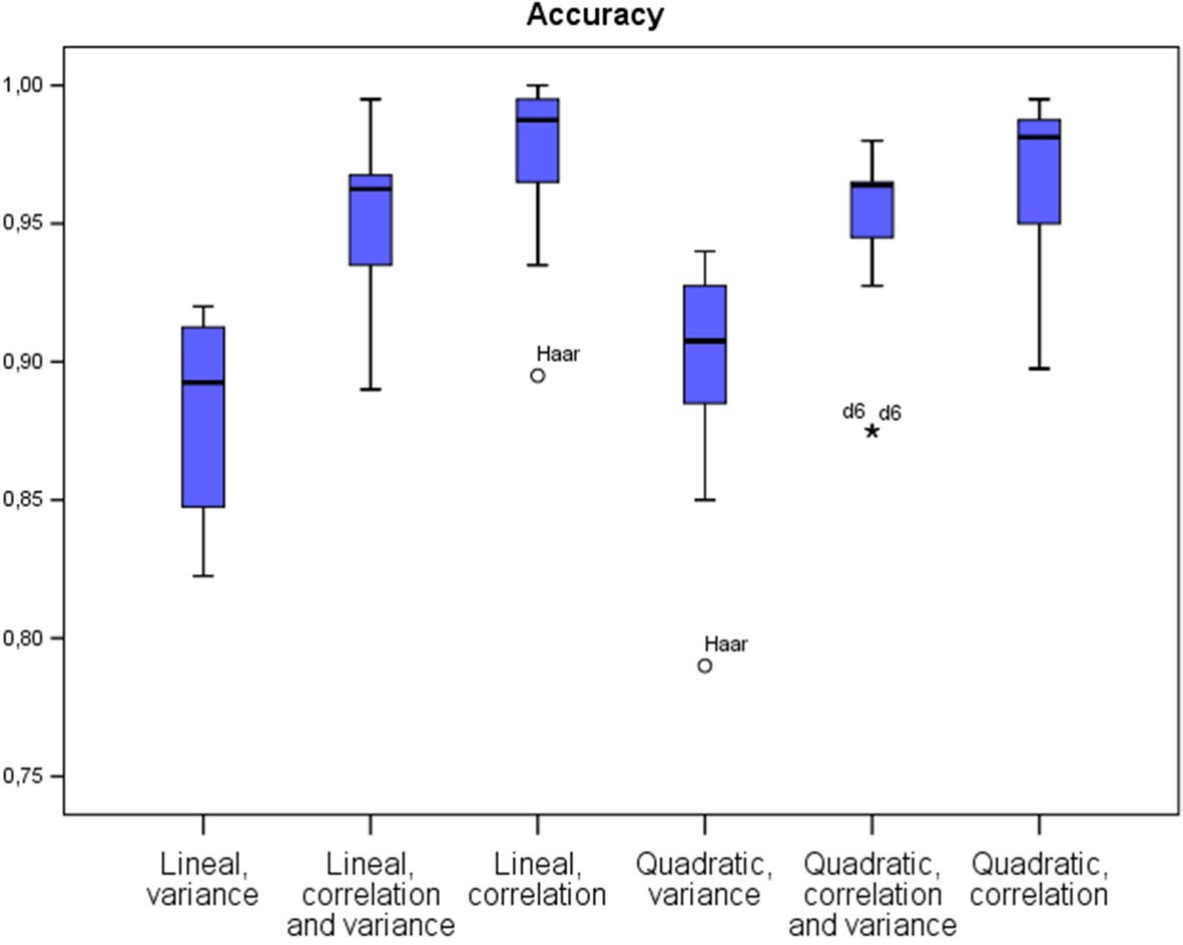
Boxplot of the accuracy of each algorithm

**Fig. 4:**
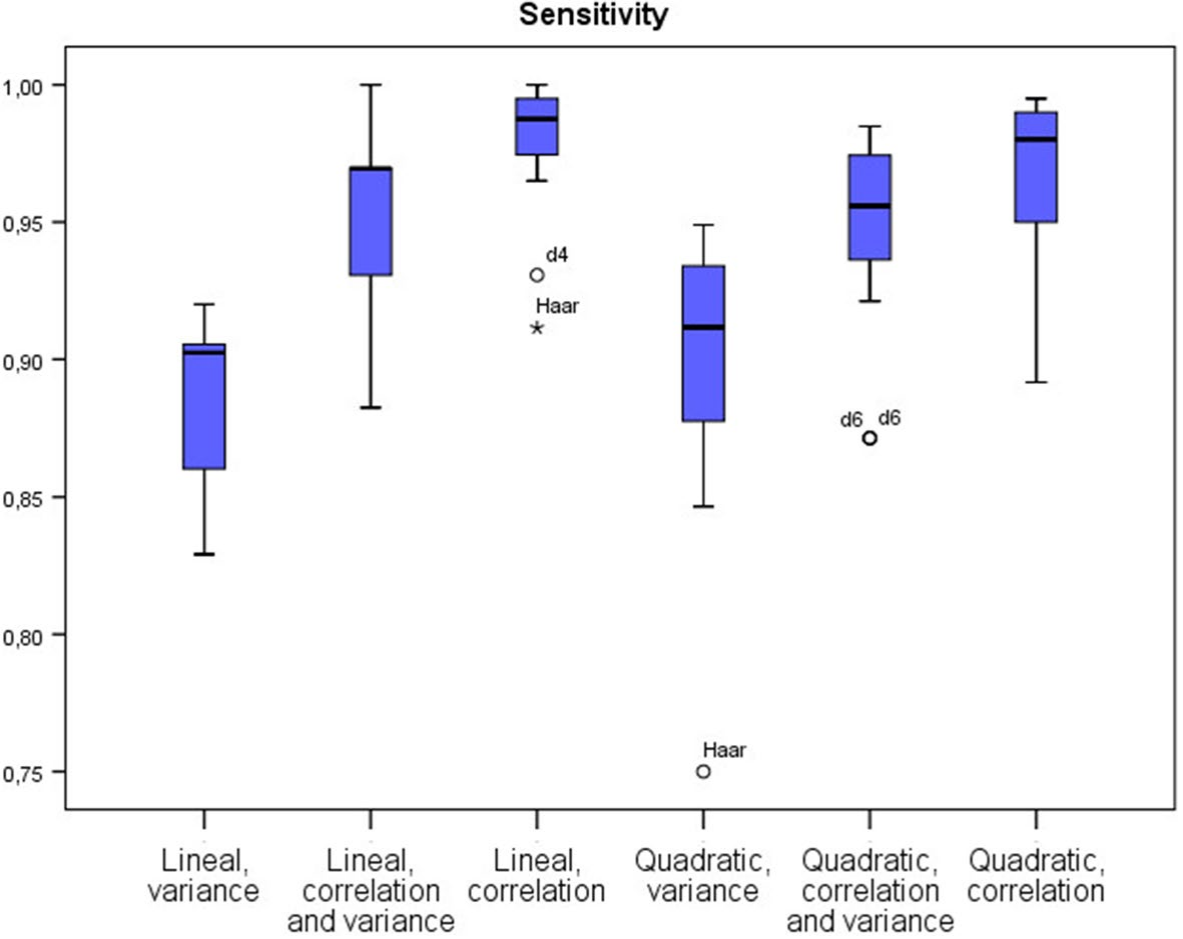
Boxplot of the sensitivity of each algorithm

**Fig. 5:**
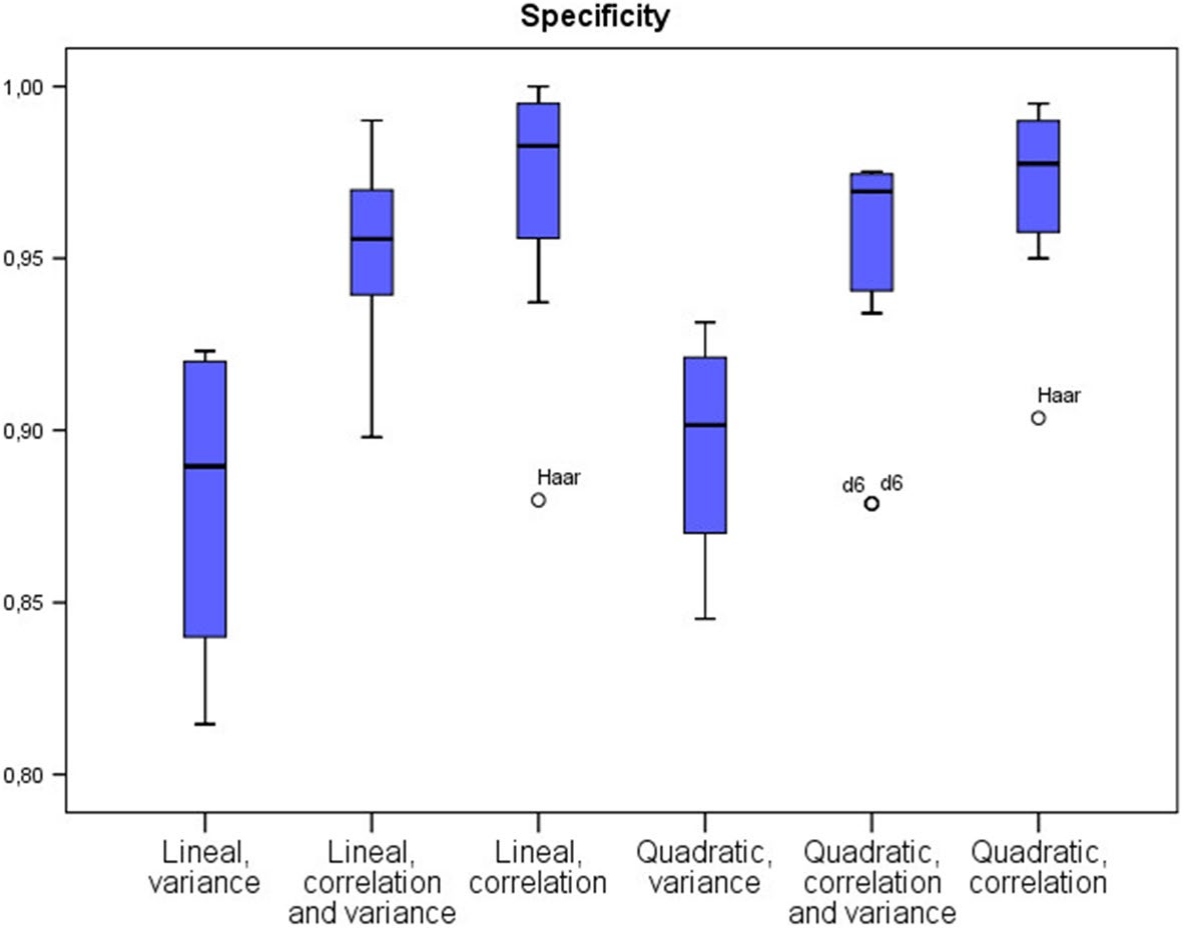
Boxplot of the specificity of each algorithm

Table 1 provides a summary of different ratios and indicators derived from the confusion matrices. Best results in terms of accuracy, sensitivity and specificity are attained using the correlation strategy under the lineal discrimination model. Furthermore, for $n=60$, where *n* is the number of variables, values of accuracy, sensitivity and specificity reached 100% for all considered instances. The quadratic discriminant under the correlation algorithm also provides good performance, but fails in considering a linear model, requiring non-linear optimization techniques. As highlighted in [21], this is not surprising, as they also found that the linear classifier was the best choice in their research framework compared with other more complex classifiers. Moreover, the advantages of a linear discriminant model compared to more complex models are the guarantees of convergence of the model parameters and the robustness against new data. Furthermore, these models are easier to interpret and explain. Other measures and their mean values can be found in the Additional file section. Concerning the wavelets considered, the best results were obtained for *la6* and *d8* (see Additional file 1 again for further details). Figures 3, 4 and 5 show the accuracy, sensitivity and specificity performance in a boxplot diagram. Also, for $n=20$ variables in the model, the poor performance of the *d6* and *Haar* wavelets can be seen.

Next subsection details an analysis based on the selected electrodes for the classification process.

**Table 1:** Average values obtained in the classification process using the following filters: Haar, d4, d6, d8, la6 and c6

	Size	Linear			Quadratic		
		Var	Var & Cor	Cor	Var	Var & Cor	Cor
Accuracy	20	0.86	0.94	**0.95**	0.86	0.94	0.94
	40	0.90	0.96	**0.98**	0.92	0.95	**0.98**
	60	0.90	0.96	**1.00**	0.91	0.95	0.99
Sensitivity	20	0.87	0.94	**0.96**	0.86	0.93	0.94
	40	0.89	0.96	**0.99**	0.92	0.95	0.97
	60	0.89	0.96	**1.00**	0.92	0.95	0.99
Specificity	20	0.84	0.93	0.94	0.87	0.94	0.95
	40	0.90	0.96	**0.98**	0.91	0.95	**0.98**
	60	0.90	0.96	**1.00**	0.91	0.95	0.99
F-Measure	20	0.85	0.94	**0.95**	0.86	0.94	0.94
	40	0.91	0.95	**0.98**	0.90	0.96	**0.98**
	60	0.91	0.95	**1.00**	0.90	0.96	0.98

The bold emphasis indicates the best result obtained for a particular size

### Electrode analysis

As the proposed method generates a large number of features, which could lead to over-fitting, it is necessary to use a method to select the most important variables. Since each of these variables can be linked to one (or two) electrodes, it is interesting to analyze which electrodes are involved in the classification process as they might indicate that they have some influence on the analyzed task. In this case, it will be analyzed which of the selected electrodes belong to the motor cortex, that was registered through 18 electrodes (out of 63).

For this analysis, we set the following parameters that provided good accuracy with a small number of variables:

- Number of variables = 20
- Features = Correlations
- Filter = *d6*
- Decomposition levels = 12
- Discriminant = Linear

With these parameter values, the algorithm achieves an accuracy of 97.25% with a reduced number of variables (20). However, as each variable references a correlation between a decomposed level of one electrode to another, each variable involves two different electrodes. This configuration includes 40 electrodes, but if we remove the repeated electrodes that participate in more of one correlation, the number of unique electrodes is 33, nine of which belong to the motor cortex.

**Fig. 6:**
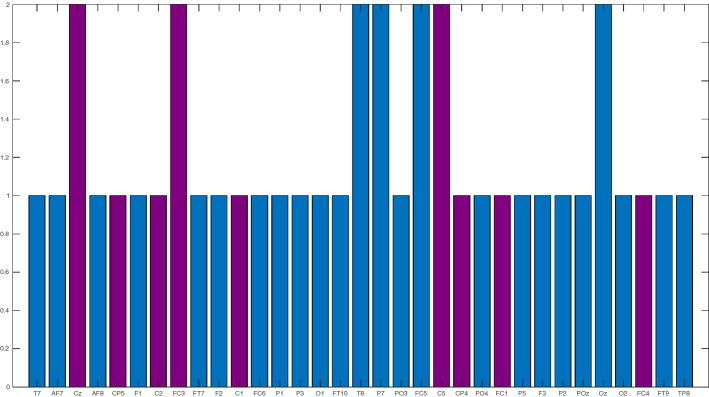
Frequency of occurrence in the correlations of the electrodes selected by the step-wise discriminant. Violet electrodes are associated with the motor cortex, while blue electrodes are associated with other parts

**Fig. 7:**
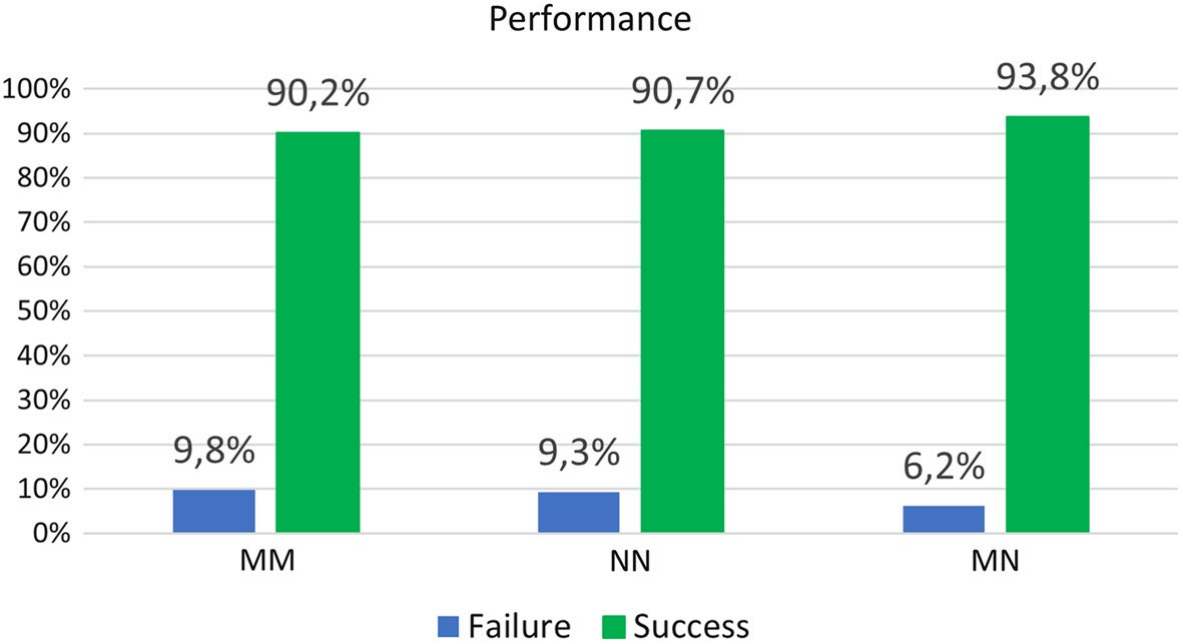
Hit ratio when combining electrodes as motor-motor (MM) motor-non motor (MN) and non motor-non motor (NN)

As can be see in Figs. 6, 7 electrodes resulted more relevant, as they are involved in two correlations. From these electrodes, four were not related to the motor cortex (T8, P7, FC5, Oz) while three of them (Cz, FC3, C5) were related to such area. It can be highlighted that if we take into account only the electrodes associated with the motor cortex, the accuracy would drop to 90%.

In order to compare the performance of the motor cortex vs the non-motor cortex electrodes and verify the electrode selection by the algorithm, we have performed a test of significant proportion differences. We must take into account, that on each experiment, we consider a Bernouilli trial where the response is “success” or “failure” in the classification. The 400 different experiments conducted allowed us to compute the average accuracy and the confidence interval for the proportion of correct classifications for each case, considering a significance level $alpha=0.05$. Figure 7 presents the frequency of success for each electrode combination. The proportion comparison test concludes that there are not significant differences in the combination of motor cortex and non-motor cortex electrodes with $p = 0.204$.

## Discussion

It is difficult to achieve good accuracy when classifying EEG signals. The aim of this work was to study and apply classification methods to achieve high accuracy. In particular, a method based on multivariate time series classification is presented and applied to motor imagery EEG signals.

The EEG data used here comes from experiments in which some subjects performed two distinct tasks. In the first task, the subjects imagine moving their hands, while in the second one, they imagine moving their feet. Our goal was to classify the different EEG signals according to whether they corresponded to feet or hands movements.

The algorithm described here performs a multiresolution analysis of the EEG electrode signals using the Maximal Overlap Discrete Wavelet Transform (MODWT). This transformation applies a decomposition at different levels, from which the lower levels capture high frequency information, while the higher levels are used for capturing low frequency information. Using the variance and correlation characteristics of these decomposed time series, discriminant analyses (both linear and quadratic) are performed over the post-processed data.

Regarding the importance of the electrodes in the proposed tasks, it has been shown that some electrodes were found relevant in the classification process are not related to the motor cortex, even when the tasks assigned during the experiment were related to the motor cortex. This seems to indicate that electrodes that are not part of the motor cortex have some kind of function in these foot and hand movements.

The good performance with *d4* wavelet makes sense, since this wavelet does not impose the condition to be almost symmetrical and considers 4 vanishing moments characteristic of EEG waves. Besides, the bad performance observed with the *Haar* wavelet could be due to the non-continuous nature of the wavelet providing a poor approximation of EEG waves. Furthermore, the linear discriminant is more accurate because the contributions of the variables in the model are directly proportional to the response.

The proposed algorithm is able to classify these tasks with an accuracy close to 100% using a limited number of variables. In some cases, with around 50 variables (out of 22176), the algorithm reaches a 100% accuracy, specificity and sensitivity. This implies that, for a particular kind of classification problems, once the subset of the most discriminant variables has been calculated, the following classifications can be optimized by using only these variables, significantly reducing the computational time.

Next, we discuss our results and compare them with previous work, starting with those whose experimental design is similar. The previous work addressed this classification problem by measuring the sensitivity of the obtained results with respect to the modifications in the applied signal extraction technique, training–testing/cross-validation routines, and hypotheses evoked in the statistical analysis [10]. They tested three different signal extraction techniques, the first one is based on Fourier analysis, the second uses parametric time series models, and the third, wavelet based techniques.

Henriques et al. found that the best average precision rate obtained with their classification method for the different subjects using the Fourier signal extraction technique was 67.6% [10], while other work only reached around 50% of accuracy [13]. We have applied a multivariate time series classification technique that robustly detects differences in the movement of healthy volunteers, reaching even up to 100% accuracy. This work has a great impact as it allowed to classify EEG data with high accuracy, and without complex training processes.

As mentioned above, although the dataset was acquired to analyze consciousness, the results obtained with this dataset could be extrapolated to the BCI domain (it has to be remarked that it consists of two different motor imagery actions; moving hands or feet). This way we can also compare our work with other proposals in this area. The results obtained with the presented methodology improve those of works that use DWT following a univariate approach to extract the features. We propose a multivariate approach that obtains, in addition, the features of the relationship between them, whereas these works only obtained features relative to a single electrode. For example, one approach propose to use DWT to obtain a frequency-domain representation, together with a Long Short-Term Memory based recurrent neuronal network, obtaining an accuracy of 87.14% [62]. Another approach uses a multistage process for feature extraction: First the EEG signals are decomposed using DWT; these decomposed signals are decomposed again into intrinsic mode functions through empirical mode decomposition; and, finally, they compute the approximate entropy of each intrinsic mode function. All these characteristics are used in support vector machine for classification, obtaining an accuracy of 95.1% [61]. The main differences are that these two works were focused on recognizing a single movement (closing the left hand) using 3 electrodes, while our proposal has been able to obtain better results (95–100% of accuracy) by splitting 2 distinct movements using 33 electrodes.

Finally, we present a comparison with other classification methods using the same dataset. Initially, we tested the performance of the MEMD method [48]. To do so, we first obtained the intrinsic mode functions (IMFs) of the signal and selected the best combination of these to reconstruct an enhanced EEG signal (testing all possible combinations). Based on that enhanced signal, the best features were obtained using Common Spatial Patterns (CSP) as proposed in [68]. Next, we tested an approach based on deep learning, specifically the adaptation of the Long Short Term Memory Fully Convolutional Network (LSTM-FCN) and the Attention LSTM-FCN (ALSTM-FCN) networks to the multivariate time series domain by means of a new squeeze-and-excitation block proposed in [39], thus creating the MLSTM-FCN and MALSTM-FCN networks, where the initial M comes from their multivariate nature. Finally, Baldan et al. [42] proposed CMFMTS, a method based on feature extraction for each individual series that composes a multivariate time series using different classifiers (support vector machine (SVM), random forest (RF) and C5.0). The CMFMTS achieves similar performance to the deep learning based method proposed in [39] but has the advantage that this method is more interpretable and it is possible to know how each feature affects the classification, contrary to the deep learning approach which is much more difficult to interpret. A comparison of these methods is shown in the Table 2.

As can be seen in Table 2, our method obtains better classification results than the rest of the methods, managing to outperform the methods based on deep learning. Specifically, for FCNs, the LSTM-FCN network is the one that obtained the best results for this dataset with 86%. The approach based on feature extraction CMFMTS achieved 90% using RF, while the MEMD algorithm obtained the worst result with an accuracy of 73%. Compared to the rest of the methods, our method has improved the classification accuracy by 5% to 10% (depending on the number of variables). However, it is even more important to note that our method is the most interpretable. This is due, on the one hand, to the reduced number of variables used for the classification, and on the other hand, to the fact that each of these variables refers to the variance of the signal of an electrode in a frequency range, or to the correlation of two electrodes in the same frequency range, which allows to easily discern which information is being taken into account by the classifier. This level of interpretability is not available in the other methods. As for neural networks, they are very difficult to interpret, and although the work of [42] has attempted to address this lack of interpretability, their model takes into account many variables which complicates its interpretability (2583 variables were used for these data, compared to the 20 variables used with our method). In addition, some of these variables do not provide new information to the classifier because there can be some overlap between the variables, which makes their interpretation more problematic. Moreover, our method is more computationally efficient, taking 1 day to obtain the best parameters for a given problem and only a few minutes with the parameters already selected, while the MEMD approach took 4 days. On the other hand, the machine-learning based methods took a similar time (16 h), however, they require high-end graphics cards to run. We used 2 Nvidia 1080 ti and we still had to reduce the size of the epochs due to memory limitations. These graphics cards are expensive (more expensive than the entire computer we used to run our algorithm) and not available to everyone.

**Table 2:** Accuracy of the different classification methods chosen

Method	Our proposal			FNCs				CMFMTS			MEMD
Configuration	20	40	60	LSTM	ALSTM	MLSTM	MALSTM	C5.0	RF	SVM	MEMD
Accuracy	0.95	0.98	1	0.86	0.82	0.71	0.78	0.89	0.9	0.79	0.73

The row entitled “Method” shows the different methods to be compared: our proposal, the alternative based on deep learning (FNCs), another alternative based on feature extraction (CMFMTS), and the traditional method (MEMD). The row “Configuration” refers to the different configurations used for each method; in the case of our proposal, it refers to the number of variables selected (taking into account only the correlations), in FNCs the particular neural network used, in CMFMTS the classifier used, and MEMD has only been applied with one configuration

## Conclusions

This paper demonstrates that the use of multivariate time series analysis techniques is an adequate tool to characterize motor imagery EEG signals, having proven that these techniques achieve higher accuracy in classifying between hand or feet movements.

Our results show that this method can be applied to different multivariate time series data with high level of success. In our work, we applied it to EEG data, but other works also obtained good results when applying DWT to ECG data [49]. This suggests that the methodology used here can be extended to other multivariate time series data, increasing the impact of our work, as the classification algorithm could be generalized to other non-clinical domain problems with multivariate time series data.

The algorithm described here (whose executable for Windows can be downloaded at https://vg-lab.es/mtsc) presents better performance considering less number of variables compared with previous proposals, being capable to improve more complex models. Moreover, the method is easily interpretable due to the reduced number of variables needed for the classifier, and that these variables are directly related to one or two electrodes in a given frequency range, contrary to deep learning based methods that are more difficult to interpret or other methods that have a large number of variables to be considered. It must be noted that large datasets involving a large number of variables may lead to inconsistent parameters derived from the over fitting caused by the intrinsic dependence derived from models dealing with higher dimensions than required.

A limitation of the method is the need to perform an empirical pre-test, similar to Figs. 1 and 2, to determine which combination of parameters yields satisfactory results for each specific task. For example, the combination that provides the best results for the motor actions used in this work may not be the most suitable for other motor actions. In addition, if we want to simplify the method so that it only calculates the selected variables instead of the entire set of variables (in our case, 55 selected variables instead of 22176 variables), a manual adaptation is required. This adaptation should be repeated for each particular task. Regarding the results obtained, it is necessary to point out that these have been produced with data that had been preprocessed to eliminate noise, artifacts and trials with errors. Therefore, the behaviour of the method has not been tested on data without this pre-processing.

Future work with other EEG datasets is needed to verify if the methodology is valid for other types of tasks, not only motor, as seems to be the case. Moreover the literature seems to indicate that other fields of application, not only EEG, could benefit from this methodology, which also remains to be verified. Additionally, for each specific experiment, knowing which variables the method selects as the most relevant for classification could provide valuable information about different aspects, areas of interest, or unexpected results, which could be useful for detecting possible lines of research as seen in the electrode analysis, where the correlation between motor and non-motor electrodes seems to be important for classification and could indicate that these relationships are important, but further research is needed. Future studies could explore, for example, if the classification process could be applied to help in diagnosis or to differentiate between a task performed by healthy or pathological subjects.

## Methods

### Experimental protocol

This section outlines the experimental design that led to the dataset used to apply the classification method. A total sample of 20 neurologically healthy adults (11 female; 9 male) with an age range of 25–66 years was considered (mean of 35.6). The EEG signals were recorded for 5.5 s with a 64-channel electrode cap., discarding the eye electrode data for discrimination purposes. The command-following experiment paradigm designed required participants to conduct 8 tasks organized in random blocks:

- Four exercises in which the patient had to imaging clenching his fist and relaxing.
- Four exercises in which the patient had to imaging toe movement–toe relaxation.

To perform the experiment, each patient had to complete 15 trials of the required exercise after the sound of a whistle. Each participant sequentially completed a total of 8 blocks presented in pseudo-random order so that no more than 2 consecutive blocks of the same type were ever completed, with 1–2 min resting time between blocks. Each block began with the auditory presentation of the task instructions for that block and required that subjects performed an action (either squeezing their right-hand into a fist and then relaxing it, or wiggling all the toes of both feet and then relaxing them.) each time a beep was heard. Subjects should perform the action as soon as they heard each beep. After 5 s of finishing the instructions, 15 tones (the beeps) are presented binaurally (600 Hz for 60 ms) with a random interval between 4.5 and 9.5 s begins. The block ends with a relaxation instruction. This experiment included 400 exercises, half of which involved hand movements and half foot movements.

The EEG signals were recorded with a digital OSG equipment with two Schwarzer AHNS amplifiers with 44 channels with a sampling frequency of 1000 Hz. The data was high-pass filtered at 0.27 Hz and no low-pass filter was applied. The post-processing stage was carried out using the Cartool software [69], specifically, the baseline was selected as the 500 ms prior to the stimulus. The data were recalculated individually based on the mean reference, and a band pass filter was applied in the frequency range 1 Hz–40 Hz. Then, an automatic precedence was applied to eliminate the trials in which an amplitude greater than 100 $\mu$V was observed in at least one of the electrodes. Finally the trials were visually inspected to remove eye blinks, movements, and muscular artifacts. The missing data due to this artifacts were interpolated using a 3D spline algorithm. To extract the signals, different techniques based on Fourier analysis, ARMA, GARCH and conditional dynamic correlation were used for noise filtering.

A complete description of the experimental design is given in [10, 13] where, among others, use the Fourier analysis technique for data extraction, which are the data analyzed in this work.

### Data analysis

**Table 3:** Confusion matrix definition

Group	Positive	Negative
Positive	Number of true positives (TP)	Number of false negatives (FN)
Negative	Number of false positives (FP)	Number of true negatives (TN)

The methodology proposed in this paper, that has been implemented in Matlab [70] and whose Windows application is available at https://vg-lab.es/mtsc), is based on the MRA of the different time series generated by each of the EEG electrodes. For this MRA, we use the DWT [71], that decomposes each signal of multivariate time series according to an assigned number of levels. From these decomposed time series, we obtain the variance of each one of them and the correlation between each level of the decomposed time series. Based on these features (variance and correlations) a discriminant analysis is performed using both a linear discriminant and a quadratic discriminant.

To measure the performance of each algorithm we summarize the information in a confusion matrix. A confusion matrix is a tool that allows the visualization of the performance of a classification model that is used in supervised learning, from a count of the successes and errors of each of the classes in the classification. This way we can check if the model is misclassifying the classes and to what extent. Table 3 resumes the confusion matrix for a binary classification:

where:

- TP is the number of correct positive prediction.
- FN is the number of incorrect negative prediction, that is, the prediction is negative when the value would really have to be positive. These cases are also called type I errors.
- FP is the number of incorrect positive prediction, that is, the prediction is positive when the value really should be negative. These cases are also called type errors II.
- TN is the number of correct negative predictions.

Several standard terms have been defined to measure the performance of a classifier in any studio where classification systems are applied:

- Accuracy is the proportion of the total number of predictions that were correct: $\text{Accuracy}=(\text{TP}+\text{TN})/(\text{TP}+\text{FN}+\text{FP}+\text{TN})$
- Sensitivity is the proportion of negative cases that were classified as positive: $\text{Sensitivity}=\text{TP}/(\text{TP}+\text{FN})$
- Specificity is the proportion of positive cases classified as positive: $\text{Specificity} = \text{TN}/(\text{FP}+\text{TN})$
- Precision is the proportion of positive predictive value: $\text{Precision}=\text{TP}/(\text{TP}+\text{FP})$

#### Feature extraction: MODWT

As mentioned, we use the discrete wavelet transform (DWT) to re-express each time series (one per electrode in our case) in a series of coefficients associated with a certain time and a certain dyadic scale [72]. This scale allows to control the sensitivity of the method to certain frequency ranges, where smaller scales provide high frequency information and larger scales provide low frequency information. For this reason, this method performs a MRA in frequency domain (and in time domain).

While DWT is a great method for performing MRA, it has certain limitations. The most important one, for our work, is that the number of coefficients are halved in each scale, which causes that this transformation fails to maintain the time invariant property of the original series and it is difficult to associate the coefficients of certain scale with the original time series. A DWT variation has been used here for solving these problems: The Maximal Overlap Discrete Wavelet Transform (MODWT). This method generates the same number of coefficients in each scale as the observations from the original time series, maintaining the time invariant property of the original time series.

Given a DWT wavelet ($h_{j,l}$) and a scaling filter ($g_{j,l}$), where *j* is the level of decomposition, the MODWT wavelet is $\widetilde{h}_{j,l} = h_{j,l}2^{j/2}$, and the MODWT scaling filter $\widetilde{g}_{j,l}$ = $g_{j,l}/2^{j/2}$. Then, the coefficients of MODWT wavelet, $\widetilde{W_j}$, and of the scaling, $\widetilde{V_J}$, at level *j* are defined as a transformation of a time series $X = {X_t, t = 0,1,2,3,..,N-1}$ (where *N* is the size of the time series) [72]:

1 $$\begin{aligned} \widetilde{W}_{X,j,t}= & {} \sum _{l=0}^{L_j-1} \widetilde{h}_{j,l} X_{t-l mod N} \end{aligned}$$

2 $$\begin{aligned} \widetilde{V}_{X,j,t}= & {} \sum _{l=0}^{L_j-1} \widetilde{g}_{j,l} X_{t-l mod N}, \end{aligned}$$

where $L_j =(2^j -1)(L-1)+1$, being *L* the size of the selected wavelet filter.

For the discriminant analysis, we use the variance of each “decomposed” signal and the correlations between them. Given a time series $x_t, t=1,...,T$, which is a realization of the stochastic process $X_t$, the wavelet variance is calculated according to equation:

3 $$\begin{aligned} V_{X,j}^2=\frac{1}{M_j}\sum _{t=L_j-1}^{T-1}\widetilde{W}_{X,j,t}^2, \end{aligned}$$

where *j* is the level of decomposition, $W_{X,j,t}^2$ are the coefficients associated with the time series $x_t, t=1,...,T$ at level *j*, and $M_j=N-L_j+1$ is the number of wavelets coefficients excluding the boundary coefficients, being *N* the sample size.

The correlation $\rho _{XY,j}$ at level j is calculated according to the equation:

4 $$\begin{aligned} \rho _{XY,j} = \frac{\sum _{t=L_j-1}^{N-1}(\widetilde{W}_{X,j,t} - \bar{\widetilde{W}}_{X,j}) (W_{Y,j,t} - \bar{\widetilde{W}}_{Y,j})}{\sqrt{V_{X,j}} * \sqrt{V_{Y,j}} }, \end{aligned}$$

where $\bar{\widetilde{W}}_{X,j}$ is the mean of $\widetilde{W}_{X,j}$.

**Fig. 8:**
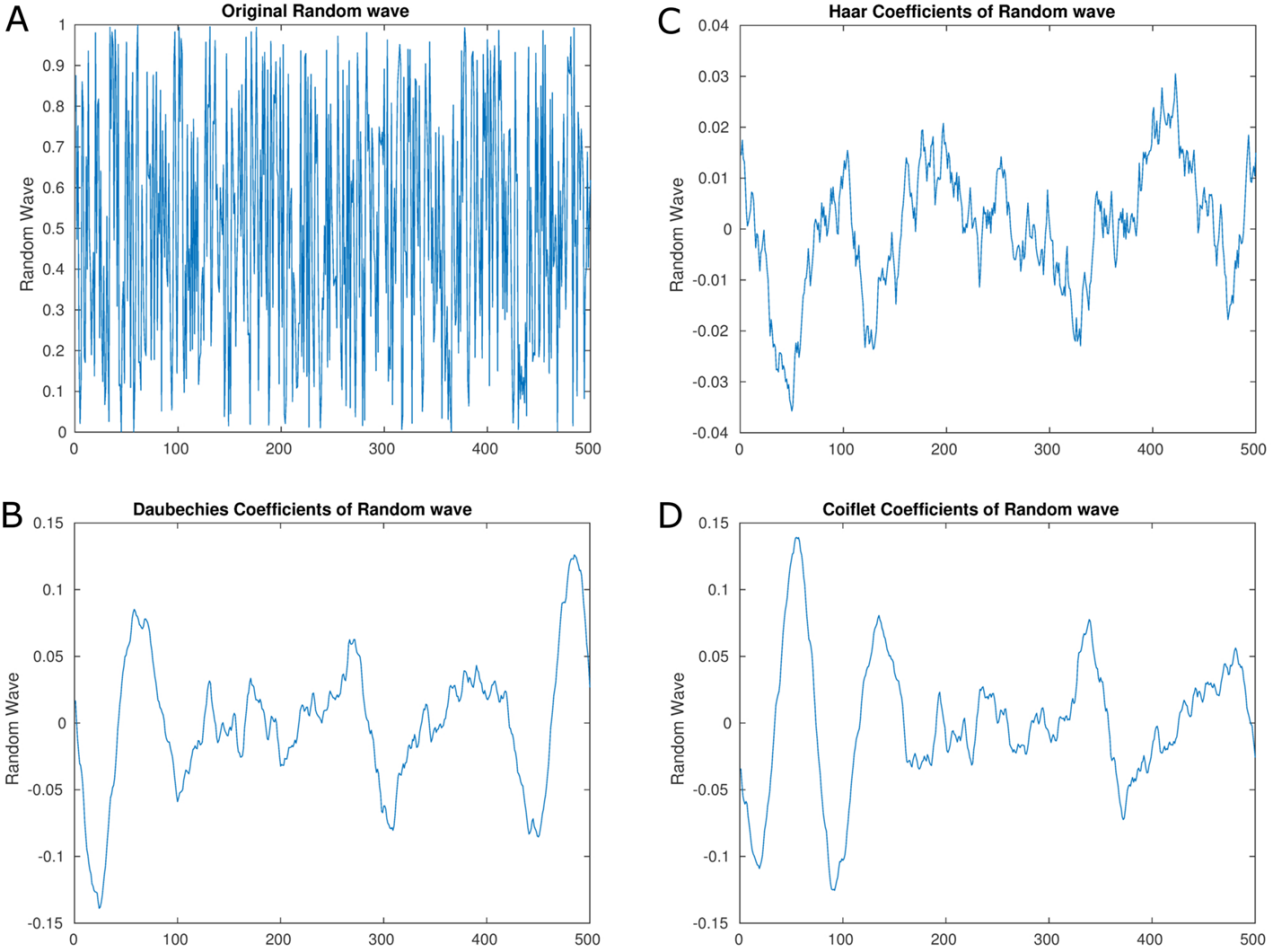
Example of different wavelets filters applied to a random wave

As discussed above, our method uses MODWT to perform a multiresolution analysis (in time and frequency) for each time series from the electrodes. This method consists of re-expressing the time series by a series of coefficients associated with a particular time and a particular level of decomposition [72] (the level of the decomposition has the form of $2^J$, where *J* is the scale of the decomposition). In this work, we use the following different wavelets families to perform the DWT analysis [71] (An example of these wavelets applied to a random wave can be seen in Fig. 8)

- Haar: This wavelet is the most basic wavelet presenting a square shape, which has the disadvantage that it is not differentiable. However, this can be an advantage for the analysis of signals with sudden changes like discrete signals [73].
- Daubechies: This family of wavelets is an extension of the Haar wavelet where each wavelet is defined by the number of vanishing moments, e.g., *db2* is a Daubechies wavelet with 2 vanishing moments. Note that the *db1* wavelet is the same that the Haar wavelet.
- Symlets: This family of wavelets is a modification of the Daubechies family that aims to obtain a family of nearly symmetrical wavelets. This property of symmetry can be useful in certain contexts where the error is less important if it appears symmetrically (e.g., in images, the symmetric error is less perceived [71]). Moreover, it is easier to deal with boundaries with symmetrical wavelets.
- Coiflets: This family of wavelets tries to maximize the number of vanishing moments, which is useful for the compression task, since it maximizes the number of coefficients close to zero which can be discarded as they do not provide much information.

In order to select the correct decomposition levels for each wavelet family, we have to take into account that *J* is the decomposition level and *N* is the number of observations (where $J\le \text{log}_2(N)$), and that, the higher the decomposition level, the more low-frequency information can be obtained. Hence, in this paper, we use the following equation to obtain the maximum decomposition level safely allowed $J < \text{log}_2(N/(L-1)+1)$, where *L* is the length of the wavelet filter used [72].

**Fig. 9:**
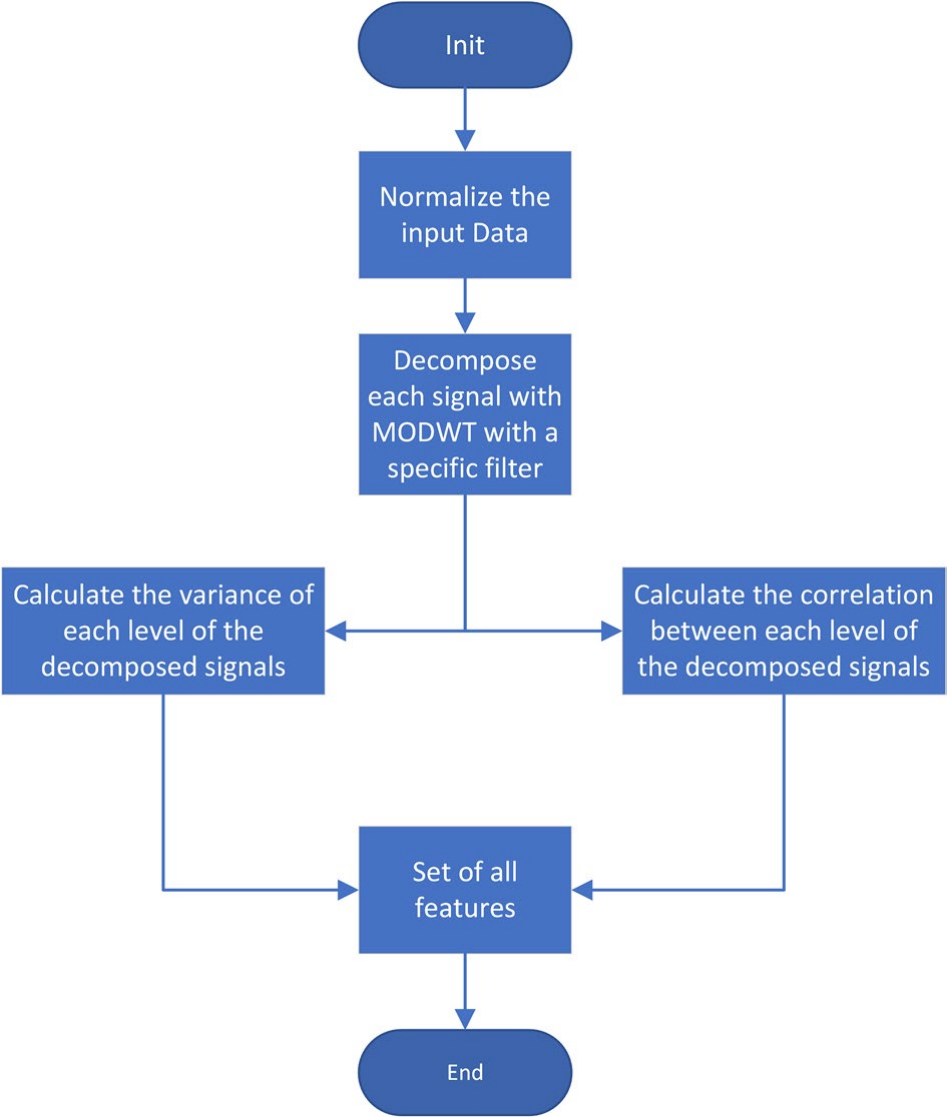
Diagram of the algorithm used to extract all the variables from the input signal

Once we have seen the decomposition process, we address the process of obtaining features for further discriminant analysis stages. The first step corresponds to a normalization process of the data by applying $\frac{(x-\bar{X})}{\sigma _x}$ to each data time series separately. Once the data are normalized, we proceed to discompose each time series as previously explained, obtaining all the decomposed signals. The total number of this signals per sample is $NDesc=NSeries * NLevs$ (in our case $NDesc=63 * 12$). From these discomposed signals, we obtain the variances of each one and the pairwise correlation of all of them, therefore: $NVar = NDesc$ and $NCors={N\atopwithdelims ()2}$. This process can be seen in Fig. 9, note that the “set of all features” contain all the variances and correlations extracted.

#### Feature selection: stepwise discriminant

**Fig. 10:**
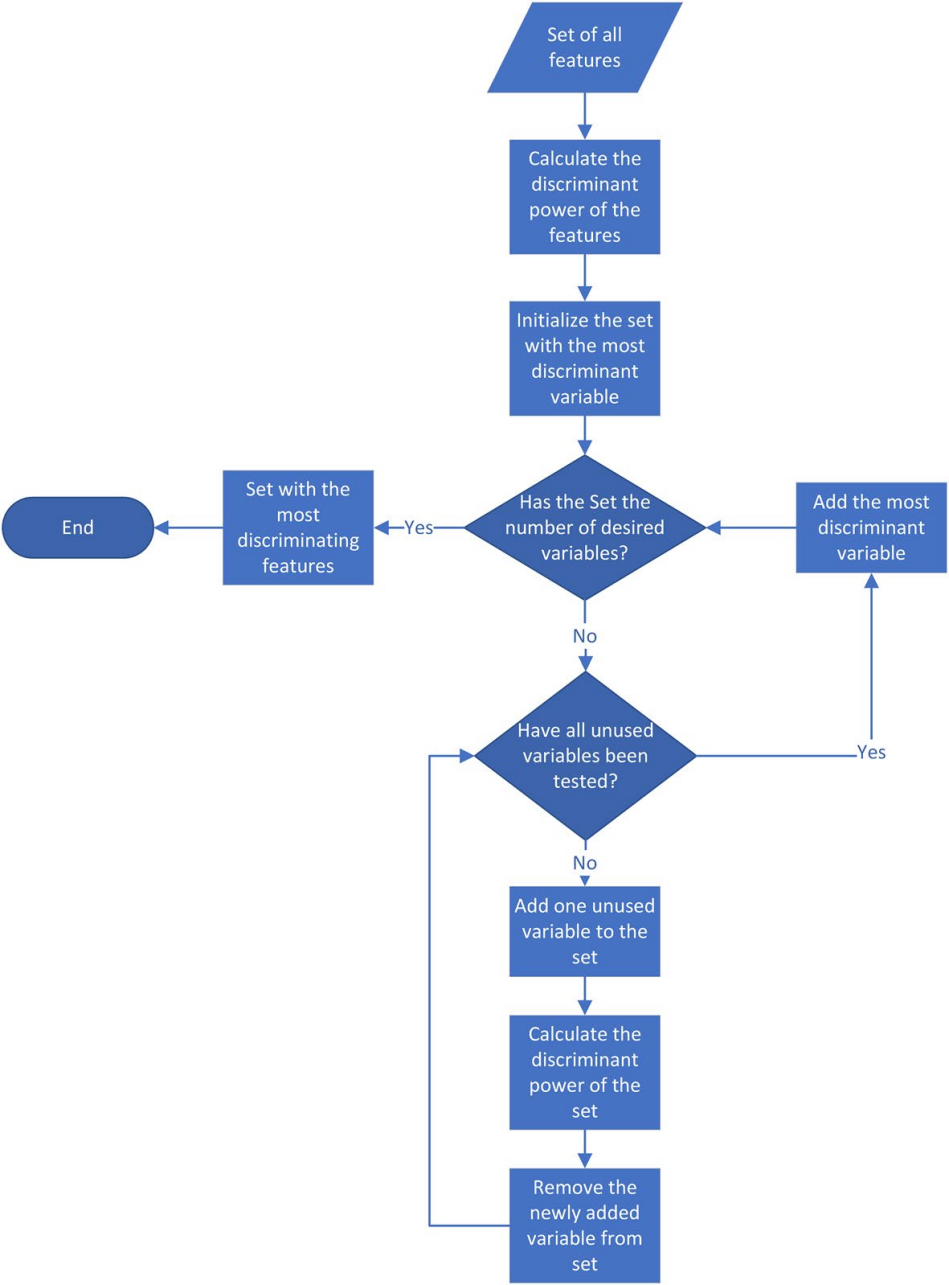
Diagram of the algorithm used to select the variables with the highest discriminant power

As can be seen, the total number of features tends to be much larger than the number of total observations, which poses a problem for the subsequent discriminant analysis. To overcome this problem, we apply an iterative algorithm to create a subset by selecting those variables (which are obtained in the previous step, so the output of the previous step is the input of this one, for more information see the Additional file 2) with the highest discriminant power. To do this, the algorithm tries the introduction of each variable in the set one by one, calculating how its addition affects the discriminant power of the set, and finally adding to the set the variable that maximizes the discriminant power (see Fig. 10). This process is repeated until the desired number of variables is reached as referred in [46]. In order to obtain the discriminant power of the set of variables, we use the Lawlley-Hotteling trace according to the equation:

5 $$\begin{aligned} V=(n-g) \sum _{i=1}^{p'} \sum _{j=1}^{p'}{a_{ij}} \sum _{k=1}^{g}{n_k (\bar{X_{ik}} - \bar{X_i}) (\bar{X_{jk}} - \bar{X_j})} \end{aligned}$$

where *n* = number of observations; *g* = numbers of groups (in our case 2); $p'$ = number of discriminating variables; $n_k$ = number of cases of group *k*; $\bar{X_{ik}}$ = mean of variable *i* in group *k*; $\bar{X_i}$ = mean of variable *i* in all groups; $a_{ij}$ = is an element from the inverse of the within-groups sum of crossproducts matrix (also called W [74]):

6 $$\begin{aligned} W_{ij}=\sum _{k=1}^{g}\sum _{m=1}^{n_k}(X_{ikm}-\bar{X_ik})(X_{jkm}-\bar{X_{jk}}), \end{aligned}$$

where $X_{ikm}$ is the value of variable *i* for case *m* in group *k*

This equation measures the distance between the centroids of the discriminated groups (i.e. the distance between the group means), but does not take into account the cohesion within the groups [74].

Based on the set of variables previously obtained, we perform a discriminant analysis using linear and quadratic discriminants. For validation purposes, we use a Leave One Out Cross-Validation (LOOCV) method [75]. This technique consists of using all the observations but one to train the classifier, using the one that was left out for testing it. This process is repeated in turn for each observation to mitigate the effect of possible anomalous observations.

## Supplementary Information

**Additional file 1.** Results supporting the box-plots of precision, sensitivity and specificity along with additional metrics and graphs are available.

**Additional file 2.** Diagram of complete algorithm.

## Data Availability

The data that support the findings of this study are available from Juan-Pablo Ortega but restrictions apply to the availability of these data, which were used under license for the current study, and so are not publicly available. Data are however available from the authors upon reasonable request and with permission of Juan-Pablo Ortega.

## Abbreviations

EEG: Electroencephalography
ECG: Electrocardiography
BCI: Brain–Computer Interface
DOC: Disorder of consciousness
fMRI: Function magnetic resonance imaging
MTSC: Multivariate Time Series Classification
MRA: Multi Resolution Analysis
DWT: Discrete wavelet transform
MODWT: Maximal overlap Discrete wavelet transform
WPD: Wavelet packet decomposition
CSP: Common Spatial Patterns
SVM: Support Vector Machine
MEMD: Empirical Mode Decomposition
IMFs: Intrinsic Mode Functions
RF: Random Forest
ML: Machine Learning
FCN: Fully Convulutional Neural Network
CNN: Convolutional Neural Network
LSTM: Long Short-Term Memory
CNN-LSTM: Convolutional Neural Network with Long Short-Term Memory
SSMVEP: Steady State Motion Visual Evoked Potential
LSTM-FNC: Long Short Term Fully Convolutional Neural Network
ALSTM-FCN: Attention LSTM-FNC
MLSTM-FCN: Multivariate LSTM-FCN
MALSTM-FCN: Multivariate ALSTM-FNC
CMFMTS: Complexity Measures and Features for Multivariate Time Series
ARMA: AutoRegressive Moving Average
GARCH: Generalized AutoRegressive Conditional Heteroscedasticity
LOOCV: Leave One Out Cross-Validation
TP: True positive
TN: True Negative
FN: False negative
FP: False Positive
Vars: Variances
Cors: Correlations
