# Supplementary figures and images for: Motor imagery EEG signal classification with a multivariate time series approach
Source: Biomed Eng Online. 2023 Mar 23;22:29. doi: 10.1186/s12938-023-01079-x (PMC10035287; doi:10.1186/s12938-023-01079-x)

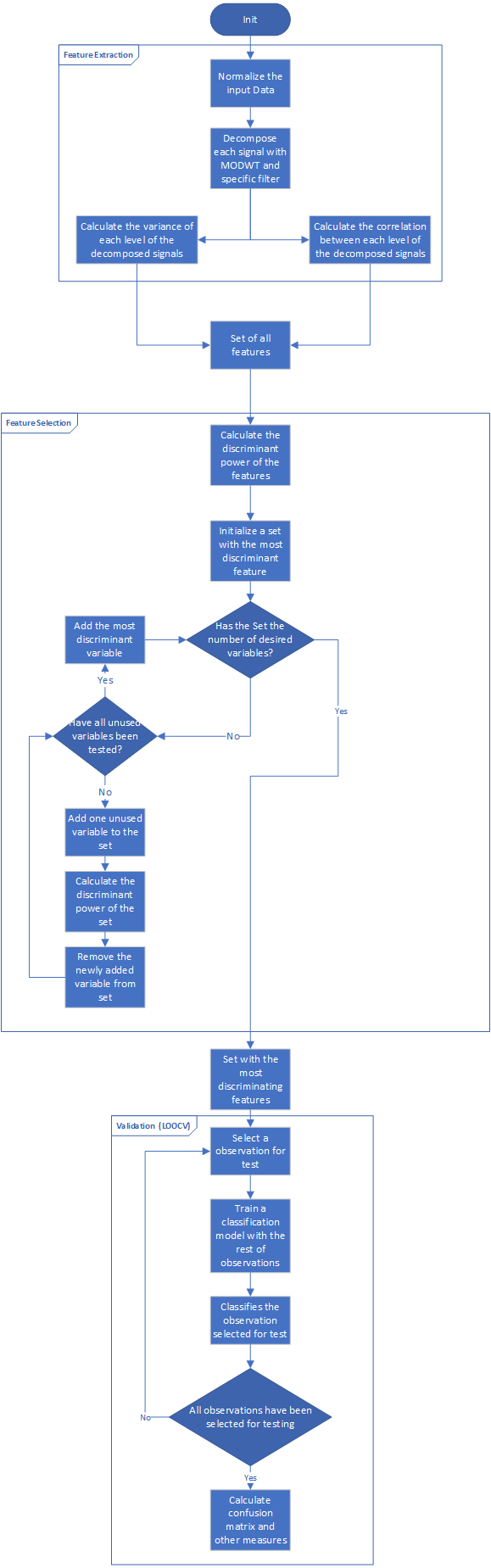

Supplement: Supplementary file 2 — Additional file 2. Diagram of complete algorithm. [file 12938_2023_1079_MOESM2_ESM.png]
